# Non-Invasive Assessment of Neurogenesis Dysfunction in Fetuses with Early-Onset Growth Restriction Using Fetal Neuronal Exosomes Isolating from Maternal Blood: A Pilot Study

**DOI:** 10.3390/ijms26041497

**Published:** 2025-02-11

**Authors:** Vladislava Gusar, Natalia Kan, Anastasia Leonova, Vitaliy Chagovets, Victor Tyutyunnik, Zarine Khachatryan, Ekaterina Yarotskaya, Gennadiy Sukhikh

**Affiliations:** 1 Laboratory of Applied Transcriptomics, Federal State Budget Institution “National Medical Research Center for Obstetrics, Gynecology and Perinatology Named after Academician V.I. Kulakov” of the Ministry of Health of the Russian Federation, 4, Oparina Street, 117997 Moscow, Russia; 2 Federal State Budget Institution “National Medical Research Center for Obstetrics, Gynecology and Perinatology Named after Academician V.I. Kulakov” of the Ministry of Health of the Russian Federation, 4, Oparina Street, 117997 Moscow, Russia; n_kan@oparina4.ru (N.K.); inter_otdel@mail.ru (E.Y.); g_sukhikh@oparina4.ru (G.S.); 3 Department of Molecular Diagnostic Methods and Personalized Medicine, Federal State Budget Institution “National Medical Research Center for Obstetrics, Gynecology and Perinatology Named after Academician V.I. Kulakov” of the Ministry of Health of the Russian Federation, 4, Oparina Street, 117997 Moscow, Russia; aa_leonova@oparina4.ru; 4 Laboratory of Metabolomics and Bioinformatics, Federal State Budget Institution “National Medical Research Center for Obstetrics, Gynecology and Perinatology Named after Academician V.I. Kulakov” of the Ministry of Health of the Russian Federation, 4, Oparina Street, 117997 Moscow, Russia; v_chagovets@oparina4.ru; 5 Center for Scientific and Clinical Research, Federal State Budget Institution “National Medical Research Center for Obstetrics, Gynecology and Perinatology Named after Academician V.I. Kulakov” of the Ministry of Health of the Russian Federation, 4, Oparina Street, 117997 Moscow, Russia; tioutiounnik@mail.ru; 6 JSC “European Medical Center”, 35, Shchepkina Street, 129090 Moscow, Russia; z.v.khachatryan@gmail.com

**Keywords:** fetal neuronal exosomes, sumoylation, neddylation, placental dysfunction, fetal growth restriction

## Abstract

The vector of modern obstetrics is aimed at finding ways to predict various placenta-associated complications, including those associated with neuronal dysfunction on in fetal growth restriction (FGR). The technology of fetal neuronal exosome (FNE) isolation from the maternal bloodstream opens up unique opportunities for detecting early signs of fetal brain damage. Using this method, FNEs were isolated from the blood of pregnant women with and without early-onset FGR, and the expression of a number of proteins in their composition was assessed (Western blotting). Significant changes in the level of proteins involved in neurogenesis (pro-BDNF (brain-derived neurotrophic factor), pro-NGF (nerve growth factor), TAG1/Contactin2) and presynaptic transmission (Synapsin 1, Synaptophysin) were revealed. The preliminary data on the expression of FNE proteins that perform post-translational modifications—sumoylation (SUMO 1, UBC9) and neddylation (NEDD8, UBC12)—were obtained. A relationship was established between altered protein expression and neonatal outcomes in newborns with growth restriction. Our study opens up new possibilities for non-invasive prenatal monitoring of fetal neurodevelopment disorders and possibilities of their correction in placenta-associated diseases.

## 1. Introduction

According to modern concepts, fetal growth restriction (FGR) is the result of altered placental function caused by a number of factors of maternal, fetal and placental origin [1]. In this condition, the fetus cannot achieve its genetically programmed intrauterine growth potential; consequently, the risk of various neonatal complications increases, including motor, and sensory deficits, and long-term cognitive dysfunction and learning problems [2,3,4]. FGR affects approximately 5–10% of pregnancies [5]. Early-onset (before 32th gestational week) and late-onset (after 32th gestational week) FGR have different clinical phenotypes and, accordingly, impact neurogenesis [6]. In general, there is a decrease in the total volume of the brain, the number of cells, and dendritic processes, and a change in the volume and structure of the cortex. At the same time, the neurological consequences are not uniform across the different areas of the gray and white matter of the brain [6]. This is primarily due to disruption of neurodevelopmental processes occurring during certain gestational periods, including neurogenesis, gliogenesis, synaptogenesis, axon myelination, cell migration and differentiation [7]. Chronic hypoxia resulting from placental insufficiency causes an adaptive response in the fetus, particularly, redistribution of cardiac output in favor of important organs, including the brain, heart, and adrenal glands [8]. This redistribution leads to the so-called brain-sparing effect, but does not ensure the brain normal development [9,10].

Doppler ultrasonography is used as the “gold standard” for monitoring the condition of the fetus during pregnancy, including assessing the brain dysfunction in FGR [11]. It should be noted that changes in blood flow resistance in the umbilical artery affect the development of the fetal nervous system in the early-onset FGR, and the dynamics of blood flow in the cerebral artery—in late-onset FGR [12,13]. While pregnancy progresses and placental resistance increases, end-diastolic blood flow in the umbilical artery decreases, ultimately leading to absent or reverse end-diastolic blood flow with a profound negative impact on the fetus [13]. However, despite the diagnostic value of Doppler ultrasonography in antenatal care, the diagnostic criteria, as well as the optimal intervals for fetal monitoring in case of growth restriction, are controversial [11]. In addition, studies of specific biomarkers in peripheral blood are limited due to the inability to assign their levels to brain pathology, as well as the relative uncertainty of their tissue origin [14].

The recently identified ability of extracellular vesicles (EVs), in particular exosomes, to cross the blood–brain barrier bidirectionally offers unique opportunities to detect early signs of brain injury [15], and the development of new methods for their isolation from the maternal bloodstream provides an innovative non-invasive platform for assessing the neurological development of the fetus in early pregnancy [16]. Previous studies have demonstrated changes in protein expression in maternal plasma-derived fetal neuronal exosomes in fetal alcohol syndrome [16,17]. It should be noted that, along with exosomal secretion, the exocytosis mechanism mediated by the SNARE complex (soluble receptors for N-ethylmaleimide-sensitive factor attachment proteins) plays a critical role in synaptic vesicle transport, maintaining neuronal function, synaptogenesis [18,19], and is also associated with a broad spectrum of neurological conditions defined as “SNAREopathies”[20]. Since the mechanisms of perinatal brain damage in FGR remain poorly understood, there are no tools for early detection of neurodysfunction in utero, we focused our study on obtaining fetal CNS-derived exosomes (fetal neuronal exosomes, FNEs) from maternal plasma. The next step was to evaluate the protein pattern of FNEs that are involved in neurogenesis, regulation of nervous system plasticity, control of neurotransmitter release and pre/post synaptic signaling in association with clinical parameters. The expression of proteins performing sumoylation and neddylation was also assessed. Regarding the last two post-translational modifications, their choice was not accidental, but based on the participation of these proteins in neuronal maturation, brain development, neurotransmission, as well as on localization and stability of pre-/postsynaptic proteins. In addition, there are no data on sumoylation and neddylation of protein targets in neurodysfunction of the brain in fetuses with growth restriction. This is the first pilot study and, to the authors’ knowledge, no studies have previously assessed protein expression in CNS-derived exosomes of fetal origin in early-onset FGR.

## 2. Results

### 2.1. Protein Expression in FNE

**Figure 1:**
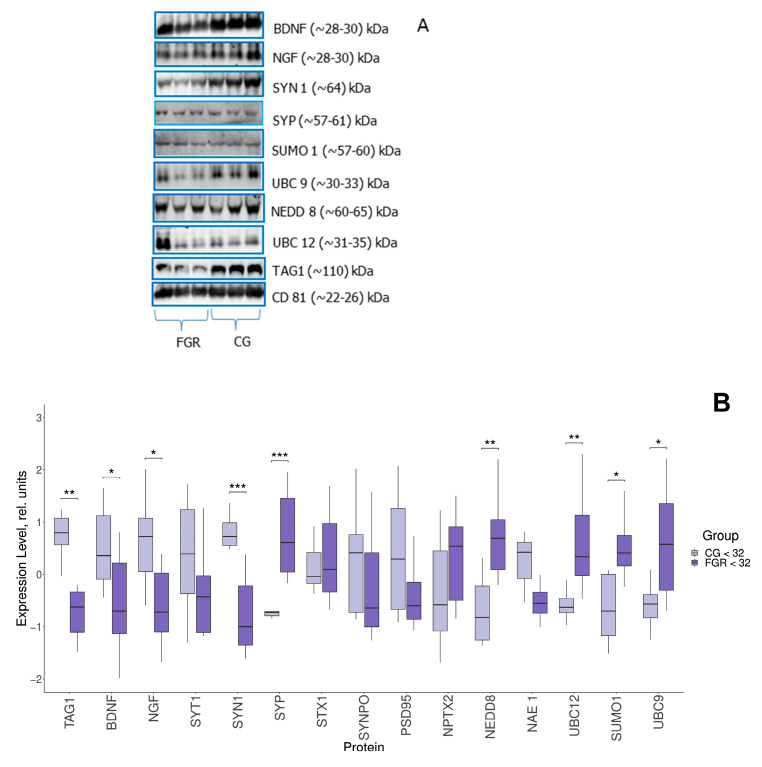
(**A**) Western blot of the membrane. The membranes represent a set of three samples from each group. (**B**) Comparative analysis of proteins in FNE from pregnant women with early-onset FGR (n = 8) and comparison group (CG) (n = 8). Data are presented in the format Me (Q1, Q3); *: significance level *p* ≤ 0.05, **: significance level *p* ≤ 0.01, ***: significance level *p* ≤ 0.001, when compared with CG. SYT1—Synaptotagmin 1, SYN1—Synapsin 1, SYP—Synaptophysin, STX1—Syntaxin 1, and SYNPO—Synaptopodin.

In the FNE samples isolated from the blood plasma of pregnant women with early-onset FGR and of the comparison group, the expression of a number of proteins involved in neurogenesis (brain-derived neurotrophic factor—BDNF, nerve growth factor—NGF), pre-/postsynaptic transmission (Synaptotagmin 1 (SYT1), Synapsin 1 (SYN1), Synaptophysin (SYP), Syntaxin 1 (STX1), Synaptopodin (SYNPO), PSD95), regulation of synaptic plasticity and excitatory synaptogenesis (Neuronal Pentraxin 2 (NPTX2)), and those performing post-translational modifications—sumoylation (SUMO 1, UBC9) and neddylation (NEDD8, APPBP1 (NAE1), UBC12)—was assessed by Western blotting. The expression of TAG1 was also evaluated in the samples (Figure 1A,B).

Comparative analysis revealed significant changes in the expression of some proteins in FNEs. In particular, the BDNF expression level (−0.7, 0.36; *p* ≤ 0.04), NGF (−0.72, 0.72; *p* ≤ 0.02), SYN 1 (−1, 0.72; *p* ≤ 0.001), and TAG1 (−0.62, 0.8; *p* ≤ 0.03) was significantly reduced in early-onset FGR in contrast to the comparison group. In this case, the expression of SYP (0.61, −0.73; *p* ≤ 0.001), NEDD8 (0.69, −0.82; *p* ≤ 0.001), UBC12 (0.34, −0.63; *p* ≤ 0.001), SUMO1 (0.41, −0.7; *p* ≤ 0.04) and UBC9 (0.57, −0.57; *p* ≤ 0.03) was increased. It is important to note that fragments of a different molecular weight were detected for a number of proteins. In particular, conjugated forms of SYP (~57–61 kDa), NEDD8 (~60–65 kDa), UBC12 (~31–35 kDa), SUMO1 (~57–60 kDa) and UBC9 (~30–33 kDa) were detected, but not the free ones. The sizes of BDNF and NGF fragments were ~28–30 kDa; such molecular weight is specific for precursors—pro-BDNF and pro-NGF, but not for mature forms. All samples were positive for CD81. It should be also noted that FNE immunoprecipitation from maternal blood samples was additionally confirmed by a significant decrease in TAG1 expression in the FGR group, indicating neurodysfunction of the fetal, but not of the maternal brain.

### 2.2. Evaluation of the Relationship Between FNE Protein Expression and Clinical Parameters in the Studied Groups of Pregnant Women

**Figure 2:**
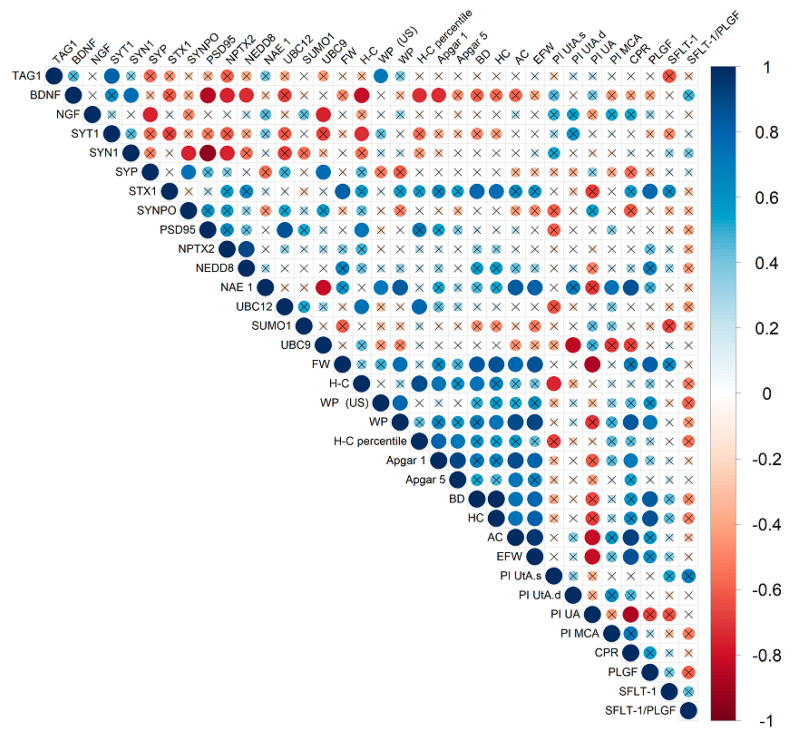
Spearman’s correlation matrix obtained from the analysis of correlations between protein expression in FNE and clinical parameters in early-onset FGR. The diameter of the circles and the color indication (according to the scale on the right) are proportional to the correlation coefficient; the fields where statistical significance of the correlation coefficients is greater than 0.05. SYT1—Synaptotagmin 1, SYN1—Synapsin 1, SYP—Synaptophysin, STX1—Syntaxin 1, SYNPO—Synaptopodin, FW—newborn weight, H-C—heel-crown, WP—weight percentile, WP (US)—weight percentile (ultrasound), BD—biparietal diameter of the fetus, HC—head circumference, AC—abdominal circumference, EFW—estimated fetal weight, PI UtA.s.—Pulsatility index of Uterine Artery (left), PI UtA.d.—Pulsatility index of uterine artery (right), PI UA—Pulsatility index of umbilical artery, PI MCA—Pulsatility index of middle cerebral artery, and CPR—cerebral placental ratio.

To assess the relationship between FNE protein expression and clinical data, we used the nonparametric Spearman rank correlation method. Since a number of indicators were not determined in the comparison group, we assessed correlations only in the FGR group (Figure 2).

Overall, significant correlations were established between differentially expressed FNE proteins and neonatal weight and length percentiles, Apgar scores, and Doppler pulsation indices, which reflect the hemodynamic state of the fetal brain. In particular, decreased TAG1 expression correlated with fetal weight percentile by ultrasound (r = 0.71; *p* ≤ 0.04). Decreased pro-BDNF expression inversely correlated with body length (r = −0.81; *p* ≤ 0.01) and length percentile of newborns (r = −0.73; *p* ≤ 0.03), Apgar 1 score (r = −0.74; *p* ≤ 0.03). A direct relationship was established with the same parameters for expression of the conjugated fragment of UBC12 (r = 0.75, *p* ≤ 0.03; r = 0.79; *p* ≤ 0.01). Interestingly, an increase in the expression of another post-translational protein UBC9 was inversely correlated with an increase in the PI of the right uterine artery (r = −0.84; *p* ≤ 0.009).

In addition to the relationship with clinical parameters, correlations between the expressions of some proteins were established. Thus, the levels of pro-BDNF and SYT1 correlated positively and with a high coefficient (r = 0.76; *p* ≤ 0.03). The expression of pro-NGF negatively correlated with the levels of SYP (r = −0.76; *p* ≤ 0.03) and UBC9 (r = −0.76; *p* ≤ 0.03). UBC9 was also associated with SYP (r = 0.76; *p* ≤ 0.03). In addition, increased expression level of NEDD8 correlated with pro-BDNF (r = −0.74; *p* ≤ 0.04).

As noted above, no statistically significant difference in expression was found for a number of FNE proteins between the comparison group and the FGR group. However, significant correlations were still found for some of them. In particular, the expression level of SYT1 (r = −0.73; *p* ≤ 0.03) and PSD95 (r = 0.73; *p* ≤ 0.03) correlated with the newborn’s length. PSD95 expression was also associated with the percentile of the newborn’s length (r = 0.71; *p* ≤ 0.05). Correlations were found between the birth weight (r = 0.81; *p* ≤ 0.02), biparietal diameter (r = 0.79; *p* ≤ 0.02), head circumference (ultrasound) (r = 0.76; *p* ≤ 0.02), and STX1 expression. The major number of parameters correlated with the level of NAE1 expression, namely: fetal weight percentile by ultrasound (r = 0.72; *p* ≤ 0.04); neonatal weight percentile (r = 0.83; *p* ≤ 0.01); abdominal circumference by ultrasound (r = 0.83; *p* ≤ 0.01); EFW (r = 0.81; *p* ≤ 0.02); PI of the fetal middle cerebral artery (r = 0.74; *p* ≤ 0.04); and CPR (r = 0.86; *p* ≤ 0.01).

### 2.3. Analysis of FNE Protein Expression Depending on the Presence or Absence of Neonatal Morbidity

**Figure 3:**
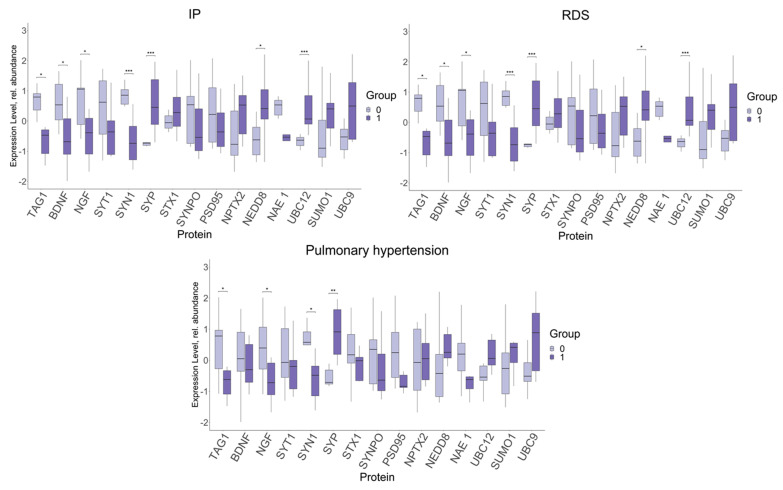
Changes in FNE protein expression in newborns with intrauterine pneumonia (IP), respiratory distress syndrome (RDS) and pulmonary hypertension (PH), depending on the presence of the disorder (1) or its absence (0). Data are presented in the format Me (Q1, Q3); *: significance level *p* ≤ 0.05. **: significance level *p* ≤ 0.01. ***: significance level *p* ≤ 0.001. SYT1—Synaptotagmin 1, SYN1—Synapsin 1, SYP—Synaptophysin, STX1—Syntaxin 1, and SYNPO—Synaptopodin.

**Figure 4:**
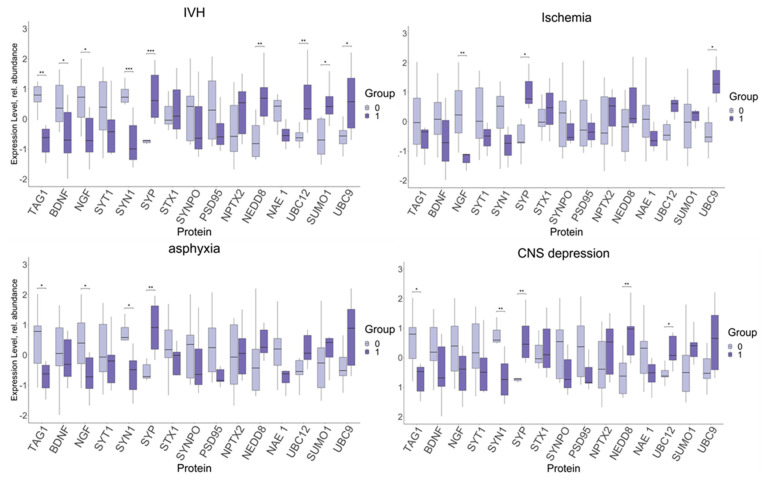
Changes in FNE protein expression in newborns with cerebral ischemia, IVH, asphyxia and CNS depression syndrome, depending on the presence of the disorder (1) or its absence (0). Data are presented in the format Me (Q1, Q3); *: significance level *p* ≤ 0.05. **: significance level *p* ≤ 0.01. ***: significance level *p* ≤ 0.001. SYT1—Synaptotagmin 1, SYN1—Synapsin 1, SYP—Synaptophysin, STX1—Syntaxin 1, and SYNPO—Synaptopodin.

**Figure 5:**
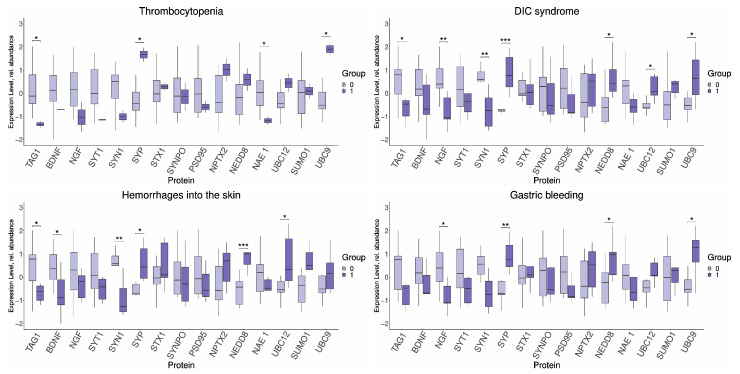
Changes in FNE protein expression in newborns with thrombocytopenia, DIC syndrome, cutaneous hemorrhage and gastrointestinal bleeding, depending on the presence of the disease (1) or its absence (0). Data are presented in the format Me (Q1, Q3); *: significance level *p* ≤ 0.05. **: significance level *p* ≤ 0.01. ***: significance level *p* ≤ 0.001. DIC syndrome is a disseminated intravascular coagulation. SYT1—Synaptotagmin 1, SYN1—Synapsin 1, SYP—Synaptophysin, STX1—Syntaxin 1, and SYNPO—Synaptopodin.

Taking into account the mentioned correlations, it was interesting to evaluate the differences in the protein expression in the FNE in the presence of neonatal complications (Figure 3, Figure 4 and Figure 5). We found a tendency for a significant change in the expression of the same set of proteins, identified by comparative analysis, of a certain disorder. Neonatal complications were combined into appropriate groups based on similar origin, such as intrauterine pneumonia (IP), respiratory distress syndrome (RDS) and pulmonary hypertension (PH) (Figure 3).

The expression of the TAG1 (−0.47, 0.79; *p* ≤ 0.04), pro-BDNF (−0.68, 0.54; *p* ≤ 0.04), and pro-NGF (−0.39, 1.06; *p* ≤ 0.04), SYN 1 (−0.74, 0.86; *p* ≤ 0.001) proteins was significantly decreased in neonates with IP and RDS, compared to the neonates without these complications. At the same time, the expression of SYP (0.45, −0.73; *p* ≤ 0.001), NEDD8 (0.41, −0.62; *p* ≤ 0.02) and UBC12 (0.07, −0.63; *p* ≤ 0.001) was increased. Similar change was observed only in four proteins, namely, TAG1 (−0.62, 0.78; *p* ≤ 0.03), pro-NGF (−0.72, 0.4; *p* ≤ 0.03), SYN 1 (−0.48, 0.58; *p* ≤ 0.02) and SYP in the presence of PH (−0.71, 0.91; *p* ≤ 0.003).

A change in the expression of neuronal proteins was found in the group of neonates with cerebral ischemia, intraventricular hemorrhage (IVH), asphyxia and CNS depression syndrome; the expression of a certain set of FNE proteins varied between these disorders (Figure 4).

Thus, TAG1 expression was significantly decreased in neonates with IVH (−0.62, 0.8; *p* ≤ 0.03), asphyxia (−0.62, 0.78; *p* ≤ 0.03), and CNS depression syndrome (−0.47, 0.79; *p* ≤ 0.01). Expression of a neurotrophin family protein, pro-NGF, was also significantly reduced in IVH (−0.72, 0.72; *p* ≤ 0.02), asphyxia (−0.72, 0.4; *p* ≤ 0.03), and cerebral ischemia (−1.13, 0.23; *p* ≤ 0.003). Moreover, a change in the expression of another neurotrophin, pro-BDNF, was observed only in the newborns with IVH (−0.7, 0.36; *p* ≤ 0.04). In addition, a significant change in the expression of synaptic proteins was found in all the above complications. In particular, SYN 1 expression was decreased in IVH (−1, 0.72; *p* ≤ 0.001), asphyxia (−0.48, 0.58; *p* ≤ 0.02), and CNS depression syndrome (−0.74, 0.59; *p* ≤ 0.007). SYP expression was significantly increased in all complications associated with brain damage and nervous system dysfunction: IVH (0.61, −0.73; *p* ≤ 0.001), ischemia (0.77, −0.7; *p* ≤ 0.03), asphyxia (0.91, −0.71; *p* ≤ 0.003), CNS depression syndrome (0.45, −0.72; *p* ≤ 0.005). In these complications, in addition to neuronal and synaptic proteins, the expression of proteins involved in post-translational modifications, sumoylation and neddylation was also altered. The increased expression of conjugated fragments of neddylation proteins NEDD8 (0.69, −0.82; *p* ≤ 0.001; 0.97, −0.62; *p* ≤ 0.007) and UBC12 (0.34, −0.63; *p* ≤ 0.001; 0.07, −0.62; *p* ≤ 0.03) was observed in newborns with IVH and CNS depression syndrome. The expression of sumoylation proteins, SUMO 1 (0.41, −0.7; *p* ≤ 0.04) and UBC9 (0.57, −0.57; *p* ≤ 0.03) was also significantly higher in IVH. The UBC9 expression was also increased in ischemia (1.28, −0.53; *p* ≤ 0.01).

In a number of clinical neonatal complications associated with hemostasis disorders, we also found significant disorder-dependent changes in the expression of a similar set of proteins with minor variations in their number (Figure 5).

In particular, the expression of TAG1 (−1.33, −0.12; *p* ≤ 0.01), SYP (1.67, −0.44; *p* ≤ 0.03), UBC9 (1.9, −0.51; *p* ≤ 0.01), NAE1 (−1.18, 0.04; *p* ≤ 0.03) was altered in newborns with thrombocytopenia. At the same time, the pro-NGF (−1.09, 0.39; *p* ≤ 0.01), SYP (0.77, −0.7; *p* ≤ 0.005), NEDD8 (1.07, −0.23; *p* ≤ 0.03) and UBC9 (1.28, −0.53; *p* ≤ 0.02) demonstrated altered expression in cases of neonatal gastrointestinal bleeding, and TAG1 (−0.47, 0.79; *p* ≤ 0.02; −0.62, 0.78; *p* ≤ 0.03), SYN 1 (−0.74, 0.59; *p* ≤ 0.002; −1.27, 0.58; *p* ≤ 0.001), SYP (0.77, −0.72; *p* ≤ 0.001; 0.44, −0.71; *p* ≤ 0.02), NEDD8 (0.41, −0.62; *p* ≤ 0.01; 1, −0.43; *p* ≤ 0.001) and UBC12 (0.07, −0.62; *p* ≤ 0.02; 0.33, −0.54; *p* ≤ 0.04), in neonates with DIC syndrome and cutaneous hemorrhage. Differences were observed in the changes in the neurotrophin expression, in particular, pro-BDNF (−0.89, 0.36; *p* ≤ 0.04) in hemorrhagic complications, and pro-NGF (−1.05, 0.4; *p* ≤ 0.005) in DIC syndrome. The expression of UBC9 was also changed (0.65, −0.53; *p* ≤ 0.04) in newborns with DIC syndrome.

## 3. Discussion

Neurogenesis is completed around the 28th week of gestation and placental dysfunction occurring during this period leads to an altered fetal neurodevelopmental trajectory associated with early-onset FGR [7]. Along with this, the degree of brain damage, birth weight, and preterm birth are significant factors influencing motor, cognitive, and behavioral functions at later age [21]. CNS-derived EV, released with certain cargo of lipids, proteins and nucleic acids of lipids, proteins and nucleic acids are an integral part of placenta-mediated mechanisms of perinatal fetal brain injury in growth restriction within the placental–fetal–brain axis [22,23]. Therefore, the altered cargo in the EV may both reflect the degree of brain dysfunction and act as potential prognostic and diagnostic markers of a pathological condition.

Under this paradigm, FNE were isolated by immunoprecipitation from the plasma of women with early-onset FGR, and the expression of several key neuronal proteins was assessed. In particular, a significant decrease in the expression of BDNF and NGF was found in FNE of fetuses with growth restriction. The proteins members of the neurotrophin family play a critical role in neuronal survival, proliferation, migration, function, and synaptic plasticity [24,25], and are highly expressed in the hippocampus and cortex during normal brain development. In addition, NGF is also present in high concentrations in the pituitary gland [26], while BDNF can be expressed in the cerebellum, thalamus, amygdala, spinal cord [25]. The latest data indicate the participation of neurotrophins in the regulation of implantation, maternal immunity, angiogenesis [27], and maturation and development of the placenta [24,28,29]. Previously, the decreased levels of neurotrophins in maternal blood have been shown in preeclampsia [30]. Another study found no significant differences in circulating BDNF levels of mothers, fetuses, and neonates with and without growth restriction, while NGF levels were decreased in pregnancies with FGR [31]. In our opinion, circulating levels of neurotrophins in either maternal or cord blood cannot adequately reflect changes occurring in the fetal brain, since they enter the blood from various organs and their secretion is influenced by numerous factors, including maternal nutrition during pregnancy [24]. Moreover, as it was previously demonstrated, the source of FNE isolated from maternal blood is the fetal brain, not the placenta [17]. In addition, we noted that FNE did not contain mature forms of BDNF and NGF, but their precursors—pro-BDNF (~28–30 kDa) and pro-NGF (~28–30 kDa), at a lower level of expression. The BDNF protein is synthesized in the form of precursor of pro-BDNF with a molecular weight of ~31–35 kDa, which is further cleaved to a mature form of 14 kDa. A significant decrease in the pro-BDNF level was shown in Alzheimer’s disease [32]. It has been shown that pro-BDNF is packaged into secretory vesicles, and once released from the cell, its functions depend on the type of target receptor it binds to [25,33]. NGF supports the development, survival and function of neurons in the peripheral (sympathetic and sensory) and central (cholinergic) nervous systems. It has previously been shown that the mature NGF is absent in the human brain [34]. Pro-NGF is secreted by many cells, including neurons and astrocytes, [35] and is released through the constitutive secretory pathway, unlike pro-BDNF [36]. Its efficiency also depends on the association with a specific receptor. In particular, pro-NGF interaction with tyrosine kinase receptors TrkA leads to cell survival, while binding to the neurotrophin receptor p75NTR causes apoptosis. It has been found that elevated pro-NGF levels lead to neurodegeneration in Alzheimer’s disease [34,37]. Considering the dual nature of neurotrophins and the fact that the physiological consequences of changes in their precursor levels are not fully understood, we suppose that pro-BDNF and pro-NGF expression in FNE promote the activation of different signaling pathways, resulting in opposite effects, and the reduced levels of neurotrophin precursors in fetuses with growth restriction are associated with neuronal and synaptic dysfunction. Interestingly, TAG1, which was used for FNE immunoprecipitation, was significantly decreased in FGR. This protein, a member of the cell adhesion molecules family, is transiently expressed on the axonal surface of some neurons during the prenatal period. In addition, it plays an important role in nerve cell migration and axon formation. TAG-1 also helps to maintain myelination of nerve fibers in the adult brain [38].

Neurotrophic factors play an important role in synapse formation in the CNS. Decreased activity has been shown to reduce synapse density in model organisms. Synapse loss alters functional connections between brain regions, leading to cognitive decline. There is some evidence that BDNF mediates synapse formation [39]. Under the influence of neuronal activity, synaptogenesis acts as a feedback mechanism to maintain neuronal homeostasis [40]. In this regard, we assessed the expression of a number of presynaptic proteins in the FNE (SYN 1, SYT 1, SYP, STX 1 and SYNPO) and the scaffold postsynaptic protein PSD95, which indirectly reflects changes in synaptic plasticity [41]. Only the SYN 1 level was found to be statistically significantly decreased in FGR. The expression of other synaptic proteins showed a tendency to reduce, but did not reach the threshold of significance. SYN 1 is known to promote neurite outgrowth, neuronal survival, functional maturation of synapses, regulation of neurotransmitter release, neurotransmission, and synaptic plasticity [42]. SYN level was found to be increased in various brain regions during oxidative stress and hypoxia/ischemia, and its release by astrocytes and glial cells is known to mediate a neuroprotective effect [43]. At the same time, a number of studies have proved a decrease in the synaptic proteins in exosomes of neuronal origin in Alzheimer’s disease [44,45]. Taking these data into account, we suppose that in fetuses with growth restriction, the reduced levels of synaptic proteins reflect aberrant neurogenesis due to placental dysfunction, and the presence of IVH, which damages the white matter of the brain and disrupts the integrity of the blood–brain barrier, exacerbates the degree of brain dysfunction. In addition, an interesting finding was related to SYP. The expression of its conjugated fragment (~59–61 kDa) was significantly increased in FNE, while no signal from the free form of the protein was detected. The structural glycoprotein SYP is one of the most common components of synaptic vesicle membranes [46]. Once synthesized, it joins other proteins such as Synaptobrevin, SYT and SNAP25 (components of the complex (SNARE)), to form synaptic vesicle membranes through mechanisms involving neuroexocytosis and beyond [47]. SYP also belongs to a family of the non-structural soluble proteins of synaptic vesicles, such as SYN1. The timing and distribution of SYP immunoreactivity has been previously shown to be specific for different structures of the neonatal brain and different terms of pregnancy [46]. Furthermore, presynaptic SYP-positive sites located before the postsynaptic PSD-95-positive sites are characteristic regions of exosomes binding with surface of neurons [48]. A study by Goetzl E. et al. demonstrated a decrease in the free SYP in neuronal exosomes in Alzheimer’s disease [44]. However, we found no evidence for the presence of the conjugated form of SYP in CNS-derived EV; this suggests that SYP in FNE may undergo certain post-translational modifications, in particular sumoylation [49]. The presence of a conjugated and possibly sumoylated form of SYP in FNE can be considered as a compensatory response to synaptic damage in FGR. Our hypothesis is supported by the data showing that SUMO1, SUMO2/3, and UBC9 co-localize in the neurons together with pre- and postsynaptic markers such as SYP and PSD95 [50] and conjugate to synaptic proteins to regulate synaptic activity [51].

Post-translational modifications play a critical role in brain development, neuronal morphology, spinogenesis, stability of pre- and postsynaptic proteins regulating synaptic transmission and plasticity, under physiological and pathological conditions [52]. Now well-known modifications sumoylation and neddylation [53,54] have been discovered more than 30 years ago; however, their functional relevance in a context of neuropathologies remains poorly understood. Both modifications represent a dynamic reversible process of covalent attachment of ubiquitin-like proteins to the target substrate, thereby changing the biochemical and/or functional properties of the latter [55]. Sumoylation is carried out by SUMO (Small Ubiquitin-like MOdifier) proteins, which have four isoforms (SUMO 1–4); their conjugation (formation of an isopeptide bond between SUMO and the target protein) is carried out by the UBC9 enzyme [56]. Deconjugation of this process is carried out by specific proteases (SENP 1–3 and SENP 5–7) [57]. SUMO isoforms differ in conjugation dynamics and responses to cellular stress [58,59]. Sumoylation acts as a molecular switch that modulates inter- and/or intramolecular interactions of protein substrates to alter their localization, stability and/or activity [51]. We found significantly increased expression of conjugated forms of SUMO 1 (~57–60 kDa) and UBC9 (~30–33 kDa) in FNE in FGR; these findings testify the presence of sumoylated proteins with different molecular weights. Therefore, the process of sumoylation of protein substrates is actively carried out in neurons, and SUMO bound to target neuronal proteins are further loaded into FNE. It should be noted that sumoylation is strictly regulated in response to neuronal and synaptic activity [60]. It has been previously shown that a number of presynaptic proteins, including SYT 1, SYN 1a, and STX 1a, are subject to binding to SUMO 1 [49,51]. In particular, sumoylation of SYN 1a is necessary for maintaining its presynaptic localization, and a decrease in its sumoylation level is associated with neurological disorders [61]. Recent data suggest that the secretion and cargo selection of EVs are also regulated by sumoylation: its overexpression enriches the amount of proteins in EVs associated with translation and transcription, while inhibition, on the contrary, reduces them. The exosomes secreted by astrocytes under conditions of increased sumoylation modulate the proteome and functional state of neurons [62]. Furthermore, the neurons subjected to transient ischemia demonstrate elevated levels of SUMO 1 conjugation [63,64]. Extrapolating the data of other authors to our study, the joint action of the increased levels of conjugated forms of SUMO 1 and UBC9 may be considered as a stimulating mechanism for the release of presynaptic proteins and neurotransmission, as well as for modification of the FNE proteome and neuroprotection under hypoxia/ischemia caused by placental insufficiency.

Neddylation, like sumoylation, carries out the attachment of neuronal progenitor cell-expressed developmental down-regulated protein 8 (NEDD8) to a specific substrate with the help of the NEDD8 activating enzyme (E1) (NAE1 (APPBP1)), the conjugating enzyme UBC12 (E2), and various ubiquitin ligases (E3) that are not fully studied. UBC12 is also unique to the neddylation pathway, unlike ubiquitin [65]. NEDD8 is 80% homologous to ubiquitin and is the most highly expressed neuronal protein in embryonic brain tissue [66]. Neddylation is critical for the maturation and stability of dendritic spines in mature neurons, neurotransmission, and synaptic plasticity [67]. At the same time, excessive activation of neddylation pathways of protein substrates leads to apoptosis, autophagy of neurons and tumor formation [65]. Furthermore, the close association of neddylation with ubiquitination through genetic variants or mutations in genes encoding ubiquitin ligase enzymes that are common to these pathways suggests its involvement in the pathophysiology of neurodegenerative diseases and autism spectrum disorders [68]. Our study revealed a significant increase in the level of conjugated forms of NEDD8 and UBC12 in FNE in the fetuses with growth restriction. Since we did not detect the free forms of these enzymes, it may be assumed that NEDD8 is secreted into neuronal exosomes in conjugation with certain protein substrates. It should be emphasized that for neddylation, in contrast to sumoylation, very few substrates have been identified. The most studied is the cullin family, which can be modified by neddylation. If the neddylation process is inhibited, the levels of cullins bound to E3 ubiquitin ligases will decrease, which will ultimately affect the normal ubiquitination process [69]. Neddylation of PSD-95 and the metabotropic glutamate receptor mGlu7 have been shown to be necessary for synaptic maturation in pre- and postsynaptic compartments [70]. Further, neddylation controls the clustering function of PSD-95 but does not affect its protein levels [66]. It was found that overexpression of activating enzyme NAE1 (APPBP1) can induce apoptosis in primary neurons, and can be inhibited by UBC12 [71,72]. Intriguingly, NAE1 (APPBP1) level was decreased in our study, but not statistically significantly. The same applies to PSD-95 level. In this context, we suppose that the increase in UBC12 in FGR blocks APPBP1 expression in fetal brain neurons and thereby contributes to the maintenance of neddylation of certain protein substrates, in particular PSD-95 clustering. On the other hand, there is evidence that neddylation suppresses Wnt/β-catenin signaling. In this case, inhibition of NAE1 expression leads to disorganization in the formation of the cerebral cortex [73]. This phenomenon is similar to the multidirectional effects of sumoylation. In summary, the data suggest that neddylation causes versatile effects depending on the conditions and binding to various substrates and the probable influence of defects in genes encoding ubiquitin ligases for binding NEDD8 to substrates. Furthermore, neddylation homeostasis can be dynamically regulated by the balanced action of enzymes involved in neddylation and deneddylation. Interestingly, the number of neddylated substrates increases along with brain development, and when neddylation is inhibited, synaptic instability and cognitive impairment are observed in excitatory neurons of the adult brain [66]. It should be highlighted that our study of the FNE neddylation and sumoylation protein expression in FGR is pioneering, and to the authors’ knowledge, no similar studies have previously been published.

Neuronal dysfunction in the perinatal period in growth restriction may lead to global neurological disorders [7,74]. Considering the altered expression of a number of neuronal proteins in FGR, we attempted to assess these data in the context of clinical characteristics of growth restricted fetuses. Interestingly, neonatal body length parameters correlated with pro-BDNF and UBC12 expression, while TAG1 and UBC9 expression correlated with ultrasound parameters—fetal weight and PI of right uterine artery. In addition, a correlation was found between neurotrophins (pro-BDNF and pro-NGF), presynaptic proteins (SYN1 and SYP) and proteins involved in sumoylation (UBC9) and neddylation (NEDD8). It should be noted that mutations in the genes of a few of the proteins under our study have been described as associated with neuropathologies. According to the OMIM database (https://omim.org/ (accessed on 7 February 2025)), a connection has been shown between the BDNF genotype (OMIM* 113505) and the phenotype of major depressive and bipolar disorders [75]. A decrease in its expression was found in Alzheimer’s, Parkinson’s, and Huntington’s disease patients. Mutations in the gene of another neurotrophin, NGF (OMIM* 162030), are associated with hereditary sensory and autonomic neuropathy. For the TAG1/CONTACTIN 2 gene (OMIM* 190197), a genotype-phenotype correlation was found for early-onset epilepsy with or without developmental delay, as well as an association with familial myoclonic epilepsy of adults [76]. In addition to the genes mentioned, correlations with various neurological phenotypes have also been described for SYP, in particular, X-linked mental retardation (OMIM* 313475) [77]. However, despite the critical role of sumoylation and neddylation proteins in the regulation and development of the nervous system, similar associations were not found for UBC9, SUMO1, UBC12 and NEDD8.

Taking into consideration the long-term outcomes of impaired neurogenesis in growth restricted newborns, we assessed the relationship with neonatal morbidity and altered proteins levels. It was found that similar pattern proteins were associated with perinatal brain damage, in particular, with IVH, ischemia, asphyxia and CNS depression syndrome. Importantly, the change in pro-BDNF level was found only in IVH, while the pro-NGF level was changed as well as in ischemia and asphyxia. These findings indirectly confirm the participation of neuronal proteins in the regulation of signaling pathways involved in multidirectional effects and pathogenesis. Interestingly, the altered expression of proteins involved in both sumoylation and neddylation was associated only with IVH. In addition, the altered expression pattern of the studied proteins was also found in a number of pulmonary and hemostasis complications.

## 4. Materials and Methods

### 4.1. The Pregnant Women Cohort and Clinical Data

**Figure 6:**
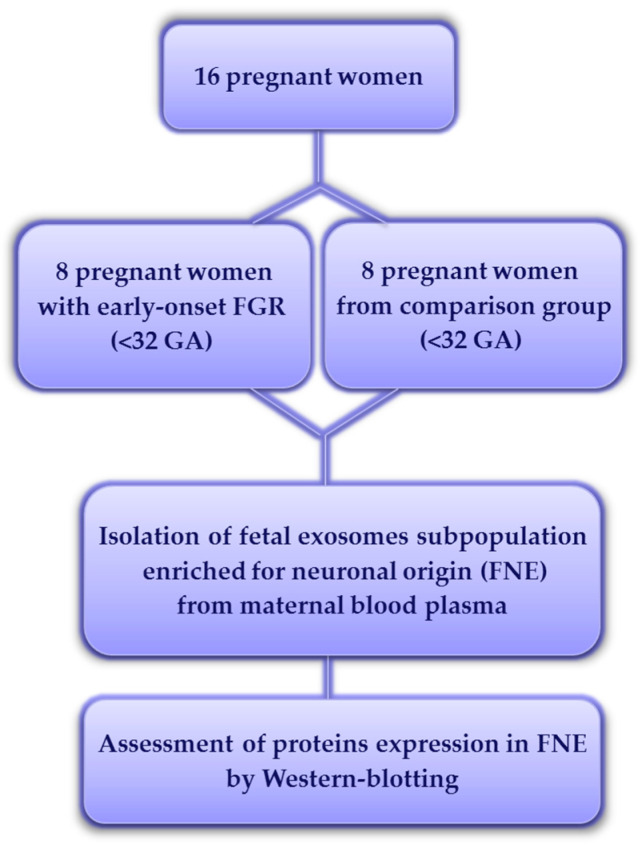
Flowchart of the study population. GA—gestational age.

This study included pregnant women receiving maternal care at the Federal State Budget Institution “National Medical Research Center for Obstetrics, Gynecology and Perinatology Named after Academician V.I. Kulakov” of the Ministry of Health of the Russian Federation. The main group were pregnant patients with early-onset FGR (n = 8; <32 weeks of pregnancy) according to the Delphi procedure [78]; the comparison group comprised pregnant women at similar terms of gestation (n = 8; <32 weeks of pregnancy) with uncomplicated pregnancy and no signs of any disorders in the fetuses accordingly to the routine antenatal tests (Figure 6). The FGR diagnosis was based on the following criteria: an estimated fetal body weight below the 3rd percentile for gestational age or the abdominal circumference less than the 3rd percentile; null and inversum diastolic blood flow in the umbilical arteries; abdominal circumference and estimated fetal weight below 10th percentile combined with PI (pulsatility index) in the uterine or umbilical arteries over the 95th percentile. In addition, amniotic fluid deficiency (oligohydramnion and anhydramnion) in pregnant women was determined using ultrasonic diagnostic criteria of oligohydramnios, namely: single deepest vertical pocket (SDVP) < 2 cm, amniotic fluid index (AFI) ≤ 5 cm. The clinical data of patients are presented in Table 1.

Blood flow in the uterine, middle cerebral and umbilical arteries of the fetus was determined using a Doppler Ultrasound System (Voluson E10, GE Healthcare Technologies, Milwaukee, WI, USA). For evaluation of the velocity curves of blood flow, the calculation of the pulsatility index (PI) was used. All studies were carried out with the informed consent of patients in accordance with the Helsinki Declaration and were approved by the Commission of Biomedical Ethics (#10, 20.10.2022) at the Federal State Budget Institution “National Medical Research Center for Obstetrics, Gynecology and Perinatology Named after Academician V.I. Kulakov” of the Ministry of Health of the Russian Federation.

### 4.2. Clinical Assessment of the Newborns and Neonatal Morbidity

The neonates were assessed in accordance with common criteria, which included birth weight and Apgar score. The stature–weight values are presented in accordance with INTERGROWTH-21st and the Fenton growth chart. A neurosonographic evaluation was performed. In addition, the neurological status of the newborns with FGR was assessed. The clinical characteristics of the newborns and neonatal outcomes are presented in Table 2.

### 4.3. Isolation of Fetal Exosomes Subpopulation Enriched for Neuronal Origin (FNE) from the Plasma Blood Samples of Pregnant Women

Plasma blood samples were collected from the study patients into VACUETTE^®^ tubes containing EDTA (Becton Dickinson, Mississauga, ON, Canada). Initially, the blood samples were centrifuged at 300× *g*, 4 °C for 20 min, and then the supernatant was centrifuged at 16,000× *g* for 10 min. and subsequently used for isolation of total maternal exosomes by ExoQuick Plasma Prep and Exosome Precipitation kit (Cat.# EXOQ5TM-1, System Biosciences, Palo Alto, CA, USA) according to the manufacturer’s instructions. Further, 250 µL of total maternal exosomes were used for FNE isolation according to protocols described in works of Goetzl L. et al. and Mustapic M. et al. [16,79]. For immunoprecipitation of FNEs was used Contactin-2/TAG1 (CNTN2). It is an axonal surface glycoprotein that is highly expressed during pregnancy [38].

Briefly, incubation was performed with 2 μg of biotinylated (Cat.# 21217 EZ-Link^TM^ Sulfo-NHS-Biotin, Thermo Fisher Scientific, Waltham, MA, USA) mouse monoclonal antibody Contactin-2/TAG1 (Cat.# MAB17141 clone 372913, R&D Systems, Fisher Scientific, USA) diluted in 50 μL of 3% Bovine Serum Albumin (BSA) (Cat.# 37525 Blocker^TM^ BSA (10×) in PBS, Thermo Fisher Scientific, USA) for 1.5 h at 20 °C mix gently by inversion. Next, immunoprecipitated antibodies were precipitated with streptavidin (Cat.# 53116 Pierce^TM^ Streptavidin Plus UltraLink^TM^ Resin, Thermo Fisher Scientific, USA) in 50 µL 3% BSA for an hour at 20 °C mix gently by inversion. The mixture was centrifuged for 10 min at 300× *g*, 4 °C. The pellet was resuspended in 100 μL of cooled 0.1 M glycine (pH-2.5). After centrifugation of the resuspended pellet for 10 min at 4000× *g*, 4 °C, the supernatant was collected, supplemented with 5 μL of 1 M Tris (pH-8) and 20 μL of 3% BSA, and then mixed with 250 μL of M-PER (Cat.# 78501 M-PER^TM^ Mammalian Protein Extraction Reagent, Thermo Fisher Scientific, USA) containing phosphatase and protease inhibitors at a concentration 3 times higher than recommended (Cat.# 78428 Halt^TM^ Phosphatase Inhibitor Single-Use Cocktail, Thermo Fisher Scientific, USA; Cat.# 11697498001 cOmplete^TM^ Protease Inhibitor Cocktail, Sigma-Aldrich, Merck, Darmstadt, Germany). The mixture was subjected to 2 freeze/thaw cycles and aliquoted for Western blotting.

### 4.4. Western Blotting of FNE Proteins

Separation of FNE proteins was performed in Tris/Tricine/SDS Buffer (12.5%). FNE samples transfer to nitrocellulose membrane (0.45 µm, Cat. # 1620115 Bio-Rad, Hercules, CA, USA) was performed using Trans-Blot SD^TM^ (Cat. # 170-3957, Bio-Rad, USA) in discontinuous buffer system for increasing the efficiency of protein transfer by semi-dry blotting. This system uses 60 mM Tris, 40 mM CAPS, pH-9.6, plus 15% ethanol in the filter paper on the anode side and 0.1% SDS on the cathode side. The membranes were blocked with 5% NFDM/TBST for 2 h for subsequent incubation during an hour at the room temperature with primary antibodies, including BDNF (UniProt: P23560) (1:1000, ab205067, Abcam, Boston*,* MA, USA), NGF (UniProt: P01138) (1:1000, SI79-01 (rabbit), Thermo Fisher Scientific, USA), Synaptotagmin 1 (SYT1) (UniProt: P21579) (1:200, sc-136480, Santa Cruz Biotechnology, Dallas, TX, USA), Synapsin 1 (SYN1) (UniProt: P17600) (1:1000, ab254349 (rabbit), Abcam, USA), Synaptophysin (SYP) (UniProt: P08247) (1:1000, sc-17750, Santa Cruz Biotechnology, USA), Syntaxin 1 (STX1) (UniProt: Q16623) (1:200, sc-12736, Santa Cruz Biotechnology, USA), Synaptopodin (SYNPO) (UniProt: Q8N3V7) (1:100, sc-515842, Santa Cruz Biotechnology, USA), PSD95 (UniProt: P78352) (1:200, sc-32291, Santa Cruz Biotechnology, USA), Neuronal Pentraxin 2 (NPTX2) (UniProt: P47972) (1:1000, ab277523 (rabbit), Abcam, USA), NEDD8 (UniProt: Q15843) (1:1000, ab81264, (rabbit), Abcam, USA), APPBP1 (NAE1) (UniProt: Q13564) (1:100, sc-390002, Santa Cruz Biotechnology, USA), UBC12 (UniProt: P61081) (1:100, sc-390064, Santa Cruz Biotechnology, USA), SUMO 1 (UniProt: P63165) (1:200; sc-5308, Santa Cruz Biotechnology, USA), UBC9 (UniProt: P63279) (1:100; sc-271057, Santa Cruz Biotechnology, USA), Contactin-2/TAG1 (UniProt: Q02246) (1:1000; MAB17141, Fisher Scientific, USA). Exosomal marker CD81 was used to assess plasma FNE levels (1:100, sc-166029, Santa Cruz Biotechnology, USA). Secondary HRP-conjugated antibodies were incubated for an hour (room temperature) in 1% NFDM/TBST (goat anti-mouse IgG-HRP (ab205719, Abcam, USA), goat anti-rabbit IgG-HRP (ab97051, Abcam, USA), sc-516102, sc-525408, sc-525409, sc-533670 (Santa Cruz Biotechnology, USA)). A SuperSignal West Femto Maximum Sensitivity Substrate Kit (Cat.# 34096, Thermo Scientific, USA) was used as a detection reagent. Densitometry was performed using Bio-Rad ImageLab 6.0 software.

### 4.5. Statistical Analysis

The statistical significance of the difference between the clinical parameters and the protein expression in the study groups was assessed by the Wilcox–Mann–Whitney test using scripts written in the R language (https://www.R-project.org/ (accessed on 7 February 2025)). Statistical data processing was conducted using RStudio version 2023.06.1 with custom scripts written in R version 4.1.1 with rstatix, corrplot and FactoMineR libraries. The Spearman nonparametric rank correlation method was used to evaluate the relationship between the protein expression and clinical parameters of pregnant women and newborns. The expression of each FNE protein was normalized to the sum of signals from all analyzed proteins in the sample and then standardized.

## 5. Conclusions

The results of this study are the first to demonstrate the possibility of identifying brain neurodysfunction in fetuses with growth restriction by the evaluation of neuronal protein expression in fetal exosomes isolated from maternal blood using immunoprecipitation. In particular, new data are presented on the protein expression pattern involved in post-translational modifications—sumoylation and neddylation, which play a critical role in synaptic plasticity and neurotransmission. It should be noted that this study is a pilot, and therefore has limitations due to the sample size. Nevertheless, the results are promising for translational medicine, since they allow identifying biomarkers for the purpose of predicting perinatal fetal brain damage in early pregnancy.

## Figures and Tables

**Table 1:** Clinical characteristics of the study patients.

	<32 GA		
	Pregnant Cohort with FGR (*n* = 8)	Comparison Group (*n* = 8)	*p*-Value
Gestational age at time of sample collection, weeks	30.4 (28.15; 30.75)	26.5 (23.75; 27.75)	0.67
Maternal age	34 (32.25; 37.75)	26.5 (23.75; 27.75)	0.01
Preeclampsia, *n* (%)	5 (62.5)	0 (0)	0.001
Ratio of placental dysfunction markers (sFLT-1/PGF; 1,5–7)	288.6 (124.8; 761.1)	NA	–
Pulsatility index of uterine artery (left) (PI UtA.s.)	1.72 (1.53; 1.89)	0.81 (0.64; 0.91)	0.001
Pulsatility index of uterine artery (right) (PI UtA.d.)	1.56 (1.42; 1.83)	0.72 (0.62; 0.77)	0.001
Pulsatility index of umbilical artery (PI UA)	1.22 (1.02; 1.99)	0.98 (0.95; 1.03)	0.12
Pulsatility index of middle cerebral artery (PI MCA)	1.67 (1.5; 1.92)	1.62 (1.47; 1.83)	0.95
Cereboplacental ratio (CPR > 1)	1.19 (0.97; 1.9)	1.77 (1.62; 1.86)	0.19
Heel–crown	31.43 (29.5; 35)	49.5 (47.25; 50.5)	<0.001
Heel–crown percentile	0.88 (0.01; 1.73)	67.14 (55.19; 79.44)	<0.001
Weight percentile (US)	0.85 (0.1; 2.5)	17.6 (16.8; 31.42)	0.001
Biparietal diameter, mm	63.95 (59.25; 65.5)	90.7 (88.58; 93.03)	<0.001
Head circumference, mm	230 (214.02; 248.12)	319.2 (312.08; 336.92)	<0.001
Abdominal circumference, mm	194 (171.52; 217.38)	322.35 (280.48; 347.55)	0.001
Estimated fetal weight, ultrasonography (EFW)	0.62 (0.56; 1.01)	2.69 (2.09; 3.21)	<0.001
Impaired fetoplacental blood flow, *n* (%)	5 (62.5)	0 (0)	0.02
Impaired uteroplacental blood flow (left), *n* (%)	7 (87.5)	0 (0)	0.001
Impaired uteroplacental blood flow (right), *n* (%)	8 (100)	0 (0)	<0.001
Null end-diastolic blood flow or absence of blood flow in the umbilical artery, *n* (%)	4 (50)	0 (0)	0.08
Oligohydramnios, *n* (%)	3 (37.5)	0 (0)	0.001
Anhydramnios, *n* (%)	1 (12.5)	0 (0)	0.001

NA—not analyzed. GA—gestational age (weeks). The data are presented in the format Me (Q1; Q3), where Me—the median, and Q1, Q3 are quartiles.

**Table 2:** Clinical state assessment of newborns and neonatal morbidity.

	<32 GA		
	Newborns with FGR (*n* = 8)	Comparison Group (*n* = 8)	*p*-Value
Birth weight, (kg)	0.7 (0.58; 1.01)	3 (2.58; 3.18)	<0.001
VLBW, *n* (%)	2 (25)	0 (0)	<0.001
ELBW, *n* (%)	6 (75)	0 (0)	<0.001
APGAR 1 min	4 (3.5; 6)	8 (8; 8)	<0.001
APGAR 5 min	6.5 (6; 7.25)	9 (9; 9)	<0.001
Neonatal morbidity:			
Intrauterine pneumonia, *n* (%)	8 (100)	1 (12.5)	0.001
RDS, *n* (%)	8 (100)	1 (12.5)	0.001
Pulmonary hypertension, *n* (%)	6 (75)	0 (0)	0.007
Hyperbilirubinemia of prematurity, *n* (%)	3 (37.5)	2 (25)	1
Neonatal asphyxia, *n* (%)	6 (75)	0 (0)	0.004
Cerebral ischemia, *n* (%)	3 (37.5)	0 (0)	0.2
IVH, *n* (%)	8 (100)	0 (0)	<0.001
Subependymal cyst, *n* (%)	3 (37.5)	0 (0)	0.2
Increased echogenicity in the PV zones, *n* (%)	2 (25)	0 (0)	0.4
CNS depression, *n* (%)	7 (87.5)	0 (0)	0.002
Anemia, *n* (%)	6 (75)	1 (12.5)	0.04
Thrombocytopenia, *n* (%)	2 (25)	0 (0)	0.4
Signs of vasodilation, increased venous outflow velocity, *n* (%)	4 (50)	0 (0)	0.07
Heterogeneous vascular plexuses, *n* (%)	2 (25)	0 (0)	0.4
Intracerebral hemorrhage, *n* (%)	1 (12.5)	0 (0)	1
Hemorrhages into the skin, *n* (%)	6 (75)	0 (0)	0.002
Gastrointestinal bleeding, *n* (%)	5 (62.5)	0 (0)	0.02
Disseminated intravascular coagulation, *n* (%)	7 (87.5)	0 (0)	0.001
NEC, *n* (%)	2 (25)	0 (0)	0.4
Muscular dystonia syndrome, *n* (%)	3 (37.5)	0 (0)	0.2
Movement disorders, *n* (%)	2 (25)	0 (0)	0.4
Hyperkinesis, *n* (%)	4 (50)	0 (0)	0.07
Tricuspid regurgitation, *n* (%)	4 (50)	2 (25)	0.6
Open ductus arteriosus, *n* (%)	8 (100)	1 (12.5)	0.001
Cardiac failure, *n* (%)	5 (62.5)	0 (0)	0.02
Enlargement of the right and left chambers of the heart, *n* (%)	6 (75)	1 (12.5)	0.03

VLBW is a very low birth weight. ELBW is an extremely low birth weight. RDS is respiratory distress syndrome. IVH is an intraventricular hemorrhage. NEC is necrotizing enterocolitis. GA—gestational age (weeks). The data are presented in the format Me (Q1; Q3), where Me—the median, and Q1, Q3 are quartiles.

## Data Availability

All data included and analyzed during the current study are an available from the corresponding author on reasonable request.
